# Endoscopic full-thickness resection of a colonic granular cell tumour using a full-thickness resection device system

**DOI:** 10.1055/a-2953-8956

**Published:** 2026-09-28

**Authors:** Luca Cossa, Elisabetta Dell'Unto, Elisabet Maristany Bosch, Georgios Kalopitas, Tu Vinh Luong, Edward John Despott, Alberto Murino

**Affiliations:** 1 UCL Institute for Liver and Digestive HealthRoyal Free Unit for Endoscopy 4965 Royal Free London NHS Foundation TrustLondonUnited Kingdom of Great Britain and Northern Ireland; 2 Department of Medicine, Surgery and Pharmacy 9312 University of SassariSassariItaly; 3 Department of Medical-Surgical Sciences and Translational Medicine, Digestive Disease Unit, ENETS Center of ExcellenceSant'Andrea University Hospital 9311 University of Rome La SapienzaRomeItaly; 4 Academic Department of Cellular Pathology 4965 Royal Free London NHS Foundation TrustLondonUnited Kingdom of Great Britain and Northern Ireland; 5 Digestive Diseases and Surgical Institute 591481 Cleveland Clinic London LtdLondonUnited Kingdom of Great Britain and Northern Ireland

Granular cell tumours (GCTs) are rare subepithelial neoplasms originating from Schwann cells. 1​ Although usually benign, rare cases of malignant behaviour have been described. 1​ ​2​ Gastrointestinal involvement is uncommon, with the oesophagus being the most frequent site. 1​ ​3​ Colonic GCTs are rare, and complete endoscopic resection may be challenging because of uncertain depth of invasion. 1​

A 34-year-old woman underwent colonoscopy for nonspecific abdominal symptoms and elevated faecal calprotectin. A 5 mm subepithelial lesion was identified in the ascending colon ( Fig. 1 ). Biopsies were non-diagnostic, showing occasional bland S100 and SOX10 positive cells within the lamina propria. The depth of invasion could not be reliably assessed. Computed tomograpghy chest, abdomen, and pelvis showed no metastasis. Following multidisciplinary discussion, endoscopic full-thickness resection (EFTR) using a full-thickness resection device (FTRD; Ovesco Endoscopy AG, Tübingen, Germany) was recommended ( Video 1 ).

**Fig. 1:**
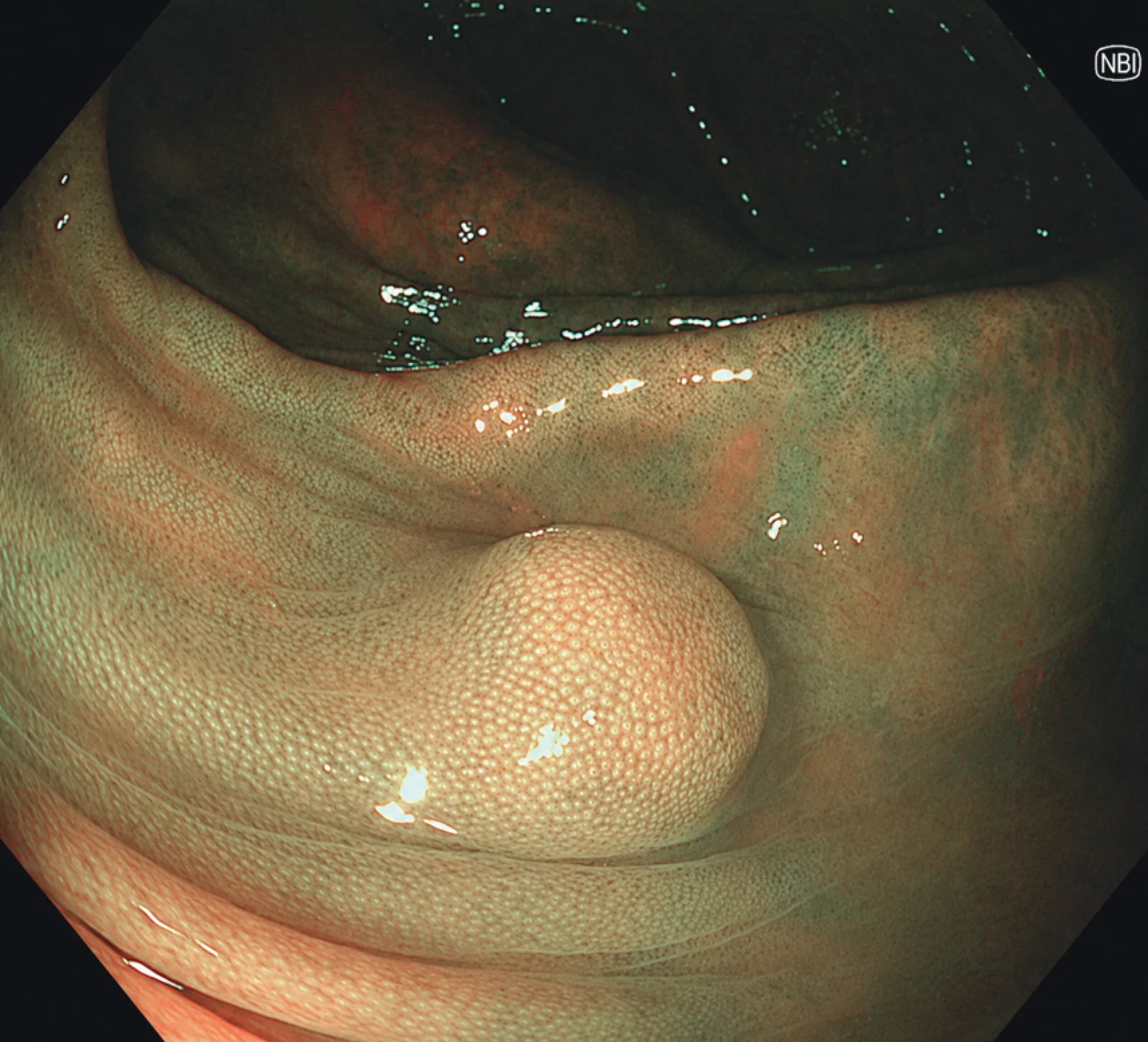
A submucosal lesion with NBI *.*

**Video 1** EFTR of a granular cell tumour in the ascending colon using an FTRD. The video demonstrates lesion marking, grasping with the Ovesco Anchor, deployment of the over-the-scope clip, en bloc resection, post-resection inspection, the resected specimen, and the histopathological findings.


A standard colonoscope was used to identify and mark the lesion margins (Forced Coagulation 1.0, ERBE Elektromedizin GmbH, Tübingen, Germany; Fig. 2 ). It was then changed to a standard colonoscope with an FTRD mounted. The lesion was grasped using an Ovesco Anchor, pulled into the FTRD cap until the marks were fitted into it and the over-the-scope clip was released, followed by successful snare closure and resection (ERBE setting HighCut 4). The colonoscope was swapped, and post-resection inspection confirmed appropriate clip deployment and resection defect ( Fig. 3 ). The specimen ( Fig. 4 ) was then pinned and sent for histopathological analysis.

**Fig. 2:**
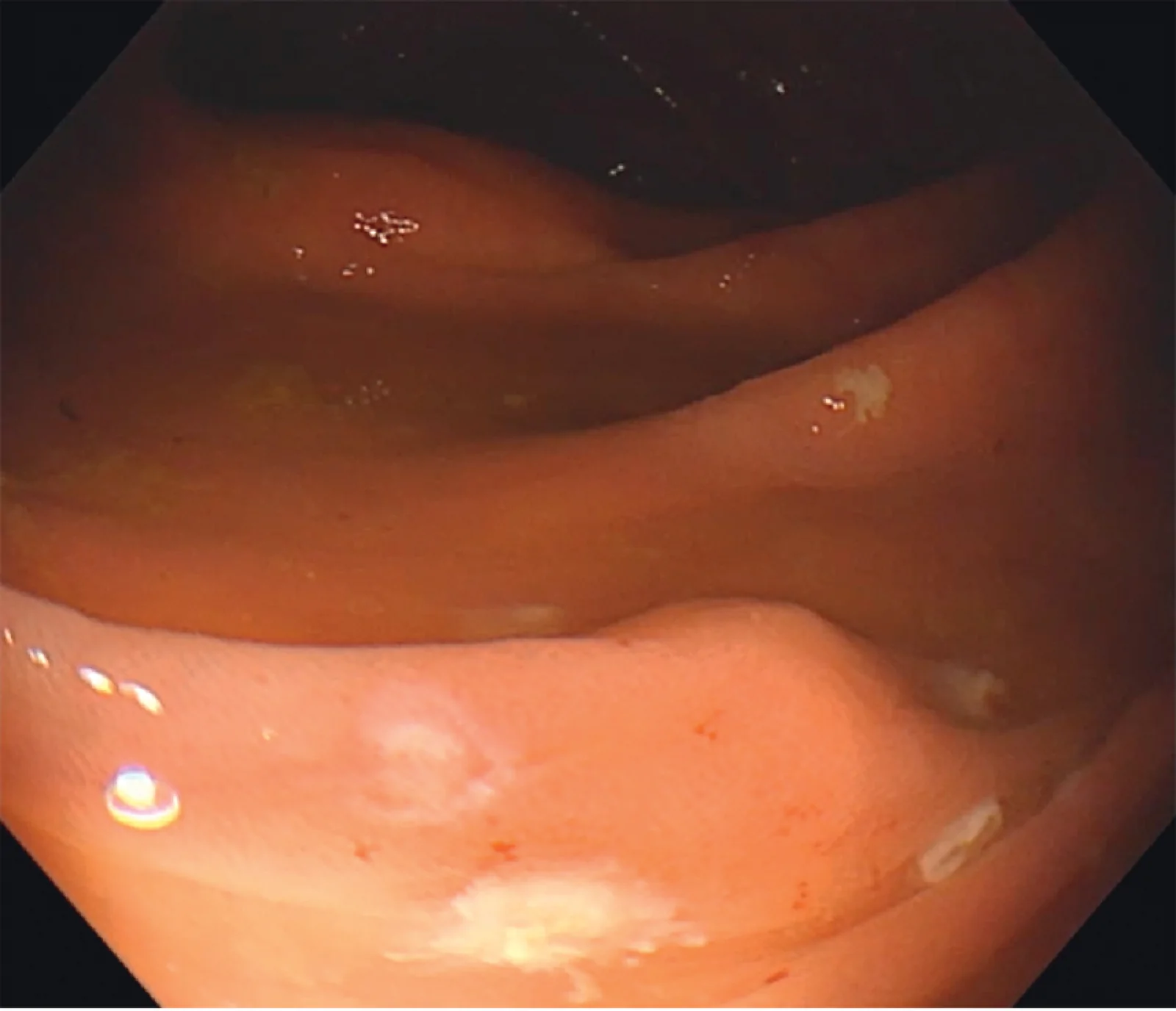
A marked submucosal lesion.

**Fig. 3:**
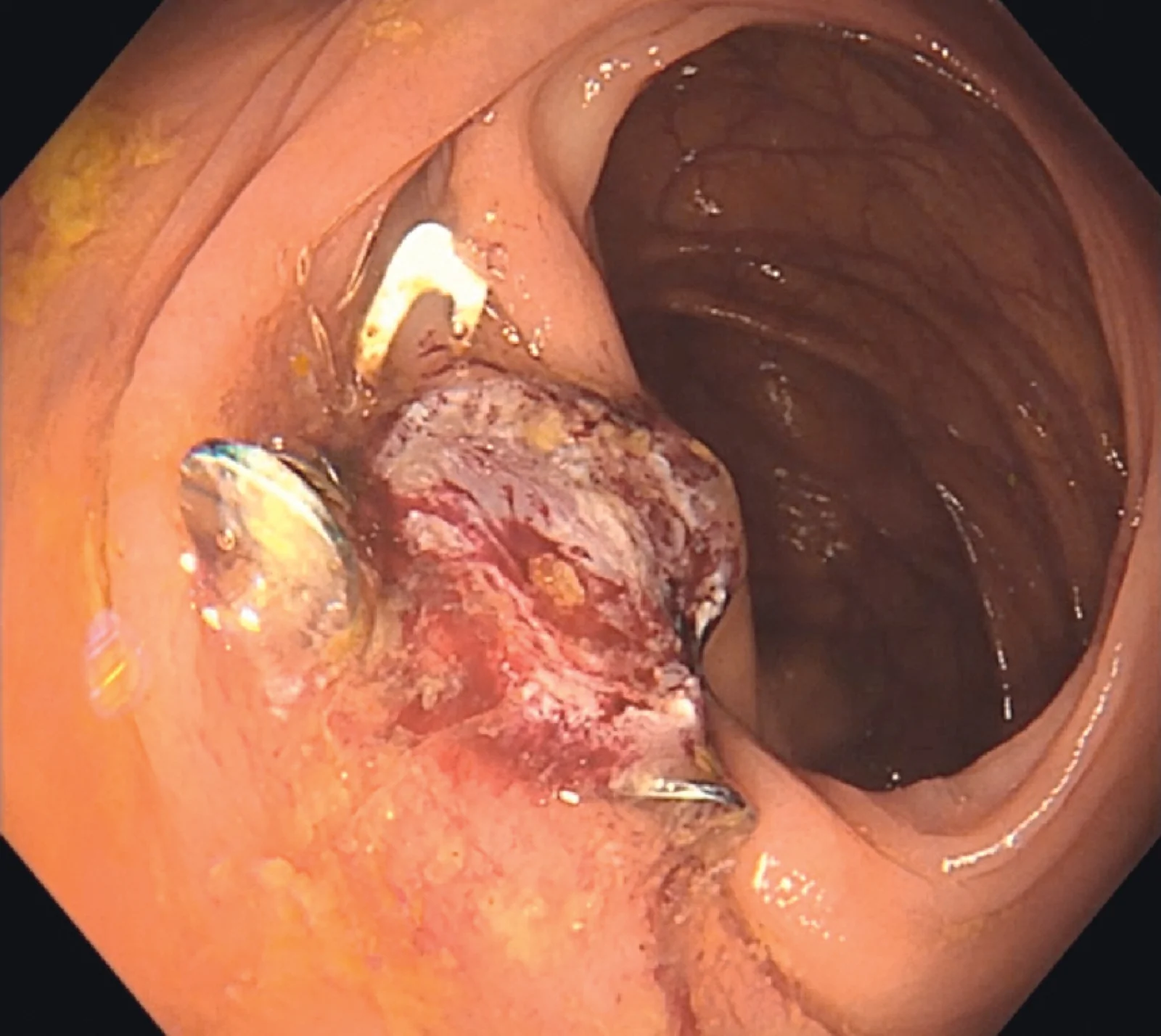
A resection site.

**Fig. 4:**
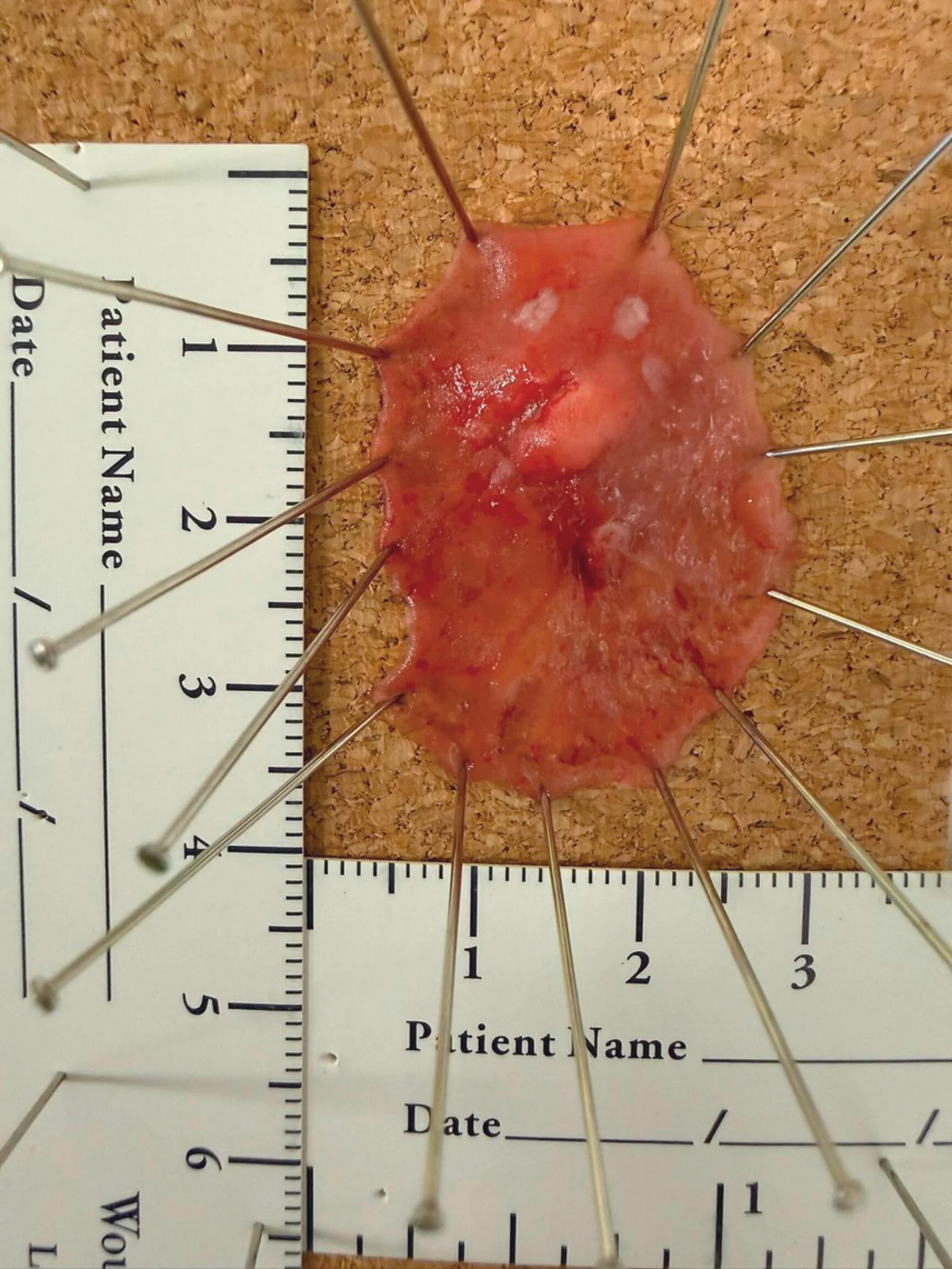
A specimen.

Histopathological examination demonstrated a 4.3 mm GCT, completely excised (R0 resection; negative resection margins) involving mainly the submucosa with focal mucosal extension. Immunohistochemistry showed positivity for S100, inhibin, CD56, CD68 (KP1), and CD68 (PGM), supporting the diagnosis ( Fig. 5 ).

**Fig. 5:**
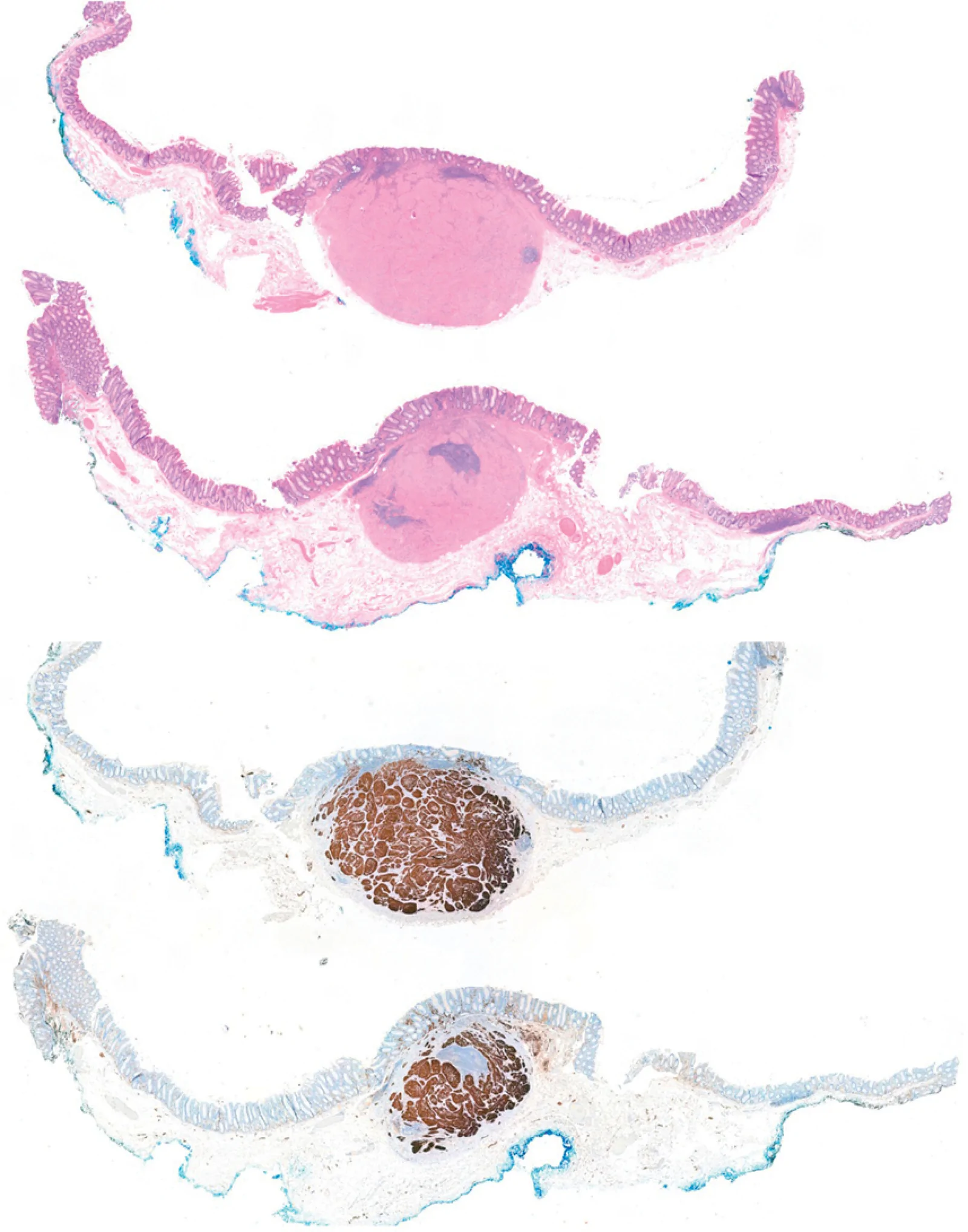
Histopathological examination of the lesion showing a haematoxylin and eosin-stained section (upper panel) and S100 immunohistochemical positivity (lower panel), consistent with a granular cell tumour completely excised.

This case highlights the usefulness and safety of the EFTR as a minimally invasive approach for the management of small colonic GCTs with possible deep submucosal involvement.

Endoscopy_UCTN_Code_CCL_1AD
